# The Book World of Medicine and Science

**Published:** 1902-05-17

**Authors:** 


### The Book World of Medicine and Science.

Pediatrics, The Hygienic and Medical Treatment of Children. By Thomas Morgan Rotch, M.D. Third Edition, rearranged and rewritten. (Philadelphia and London: J. B. Lippincott Company. 1901. Price 25s. net.)

It is seldom that we meet with a book which is so universally satisfactory throughout as is this work on pediatrics, and its uniform excellence is all the more striking when one considers the wide range of subjects which are dealt with by the author. A most important feature of the first division of the work is the careful description given of the normal development of the child. It is pointed out with great force that a knowledge of the different stages of development and of what is normal to the child at each of these stages is really the alphabet of our knowledge of children's diseases, an alphabet which when once thoroughly mastered will enable us to read the otherwise obscure language presented to us for translation by the various diseases of early life; for in children disease does not always mean a pathological change in the tissues, it may indeed mean merely a retardation or arrest of development. The reader will find that by aid of much careful description, greatly assisted by many admirable illustrations, he is thoroughly instructed, as a first step, in the conditions met with in the normal infant at term and with the normal process of the child's development in after years. All kinds of details are entered into, even to the extent of giving illustrations of the garments in which the new-born infant will be swathed by the nurse, and the second set of clothes in which it will be dressed when it is "shortened." We merely mention this to indicate the thoroughness and completeness with which Dr. Rotch deals, with every influence which may make for or against the health of the child. His remarks about nursery-maids are not without interest. "The idea that the child should be taken care of by an old experienced nurse is," he says, "a vicious one. The experience of nurses is, as a rule, that of ignorance rather than intelligence. Every mother, as she is presumably more intelligent than the nurse whom she employs, and is surely more interested in the welfare of her child, should personally supervise and unhesitatingly investigate all that the nurse does to the child. The nurse's ideas as to what is needed for the child's hygienic surroundings, food, and clothing, can well be dispensed with. The mother, learning from the physician what is best for her child, should give her directions to the nurse and see that these directions are strictly carried out. A nurse between the ages of 20 and 35 is preferable to one who is younger or older." There is no doubt that there is some common sense in this, but we must improve the modern mother before we abolish the modern child's nurse. Some mothers know so little about their babies that they seem almost afraid to touch them when they are ill.

The chapter on feeding is quite an essay by itself, and as might be expected from such an authority is full of useful advice including very careful directions for producing and prescribing the various forms of modified milk which may be required. Dr. Rotch speaks very strongly in favour of the necessary "modification" being done in a properly equipped laboratory rather than at home. A consideration of premature infants and their management, and of diseases of the new-born, usefully occupy other chapters, and then we come on the 326th page to the consideration of the various diseases of children. These are discussed in great detail, the descriptions of the conditions met with are good, and the details given as to treatment are practical and useful. Illustrations, many of them coloured and some of them most admirable, are freely introduced and the author deserves the highest praise for the manner in which he has presented his subject to his readers. It must be understood that the book is a *medical* one, the opinion of a surgeon being advised whenever surgical questions crop up; indeed, in no other way would it have been possible to limit the size of the book to the 1,026 handsome pages which it contains.

Hospital Sketches. By Lucas Galen. (London: Grant Richards. 1902. 8vo. 1s.)

These sketches have many of them appeared before in the *Pall Mall Gazette and Budget*. They are obviously the work of one who has laboured as an out-patient physician at a children's hospital, and of one in full sympathy with the sorrows of his little patients, while the author is alive to the negligence, ignorance, and vice which so largely fills such out-patient rooms with suffering offspring. The ready humour and the phonographic memories of the language heard in such places remind us at once of Mr. F. Anstey's *Voces Populi*. We select an example merely because of its short length:—

*Doctor*: "That's measles."

*Mother*: "Yes, I thought so, because, you see, I've got four other children down with measles at home."

*Doctor*: "Did you say so when you came here?"

*Mother*: "No."

*Doctor*: "You know measles is catching, don't you?" (No answer.)

*Doctor*: "Then you've been sitting here all morning dealing measles all round to a crowd of other patients."

*Mother* (doggedly): "How was I to know it was measles?"

If the author's satire is at times biting, when he touches on the constant abuse of hospital charities by the well-to-do, on the brutalities inflicted on ailing children by an unelastic educational administration, or the cruelties practised by foster-mothers, his abundant warm sympathy for little children is shown in the "Little Pelican" and "Sympathy." Every student, nurse, house surgeon, or other person who has ever entered a hospital should buy, read, and hand on this little volume to every patron, subscriber, or other person who has never set foot within a hospital doorstep. There is amusement, profit, and knowledge of mankind, womankind, and child-kind on every page.

Manipulation (or Massage). By John Andrews Peters. (Newcastle-on-Tyne: Mr. Longhurst. 1901.)

This is a book on massage as practised by its author. We have tried to find something useful in its pages, and if we have failed that is not our fault. It is filled with uninteresting trivialities. No one can really care to know that somebody who was brought up to business pursuits picked up the trick of massage from an old nurse, on the strength of which he ultimately obtained a fine "practice" and died worth a lot of money; that he parted with his "practice" to someone else, and that he in turn to others, and that in this way valuable "practices" were established in all quarters of the globe. Nor can it be important for the world to know (except so far as the world contains potential patients) that the writer also cultivates this art; and as to his pathological speculations and his cases, all one can say is that they will probably serve their purpose when the book falls into the hands of an invalid. There are far too many of such books.

